# Reevaluating the Utility of Maxillary Sinus Opacification as a Screening Tool for Facial Bone Fracture a Decade After Its Original Analysis

**DOI:** 10.7759/cureus.487

**Published:** 2016-02-09

**Authors:** Vadim Grechushkin, Konstantin Boroda, Ammar Chaudhry, Jason Eisenberg

**Affiliations:** 1 Radiology, Massachusetts General Hospital; 2 Internal Medicine, Albert Einstein College of Medicine; 3 Radiology, Stony Brook University; 4 Neuroradiology, Johns Hopkins University School of Medicine

**Keywords:** facial trauma, sinus fracture, facial bone ct, head ct, maxillofacial trauma, clear sinus sign, sinus opacification, maxillary sinus

## Abstract

In 1997, Lambert and colleagues demonstrated that the absence of sinus fluid on head CT essentially excludes a fracture involving the sinus walls. Our purpose is to reevaluate this association utilizing the current standard of imaging technology. With improved image resolution, we aim to reassess whether the sensitivity and specificity of the “clear sinus sign” are improved or worsened. Furthermore, the current standard of care is to obtain a CT of the facial bones along with a head CT when facial trauma is suspected, so we also analyzed the association of the "clear sinus sign" with nasal bone and mandible fractures. We identified 629 facial bone CT scans performed on adult patients in the emergency department between July 2012 and May 2013. They were retrospectively analyzed by three reviewers for the presence of facial bone fracture and/or fluid opacification of at least one paranasal sinus (as defined by either complete sinus opacification or an air-fluid level - circumferential mucosal thickening was considered the absence of fluid). We found that sinus opacification was 98.8% specific for facial bone fracture but only 44.7% sensitive. However, for complex facial fractures, such as zygomaticomaxillary complex, orbital, and sinus fractures, the lack of sinus fluid is significantly more sensitive at 91%. Therefore, our results for complex facial fractures are congruent with those of the previous studies conducted by Lambert, et al. and Lewandowski, et al. However, we also demonstrate that sinus opacification is not specific for nasal bone or mandibular fractures.

## Introduction

Maxillofacial trauma is potentially life-threatening due its association with spinal and intracranial injuries. With improving CT technology, multi-detector CT has become the standard of care for evaluation of patients with acute facial trauma. Full body CT is usually performed along with head CT as approximately half of maxillofacial injuries are associated with multisystem trauma [1-4].

There are approximately twelve million head CT scans performed each year and this number has been steadily rising [5]. The high volume of emergency CT scans places time pressure on interpreting radiologists, who are often responsible for multiple concurrent examinations of an acute trauma patient.

**Figure 1:**
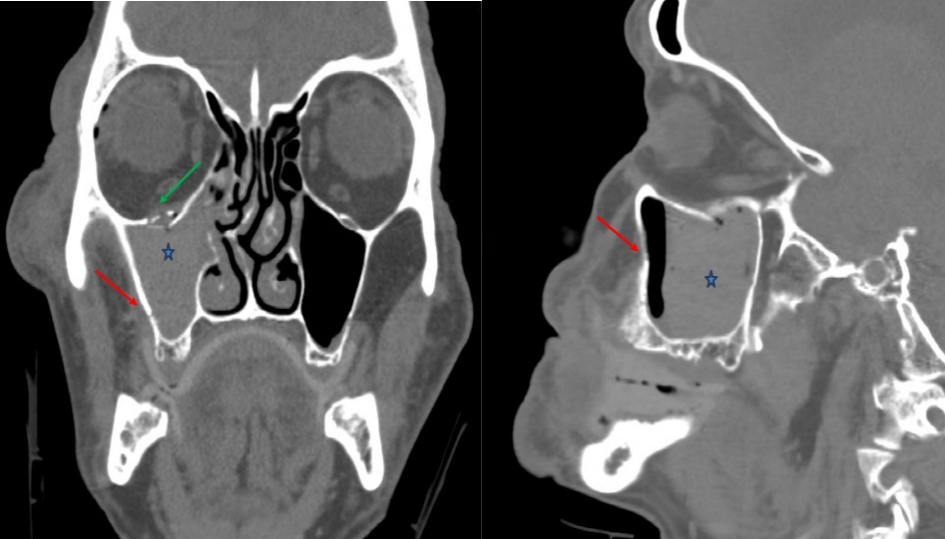
Example head CT image with maxillary sinus opacification Coronal (left) and sagittal (right) non-contrast head CT images. Example of a typical fracture involving the right orbital floor (green arrow) and medial maxillary sinus wall (red arrow), which is associated with resultant hemorrhage and an air-fluid level in the right maxillary sinus (blue star).

In this study, we aim to analyze the validity of the “clear sinus sign”, which states that the absence of sinus fluid or opacification can be used to exclude the presence of a fracture, allowing for rapid triage and diagnosis. Lambert and colleagues first validated the “clear sinus sign” in 1997 and demonstrated that the absence of fluid has a very high negative predictive value and essentially excludes a fracture involving the sinus walls [6]. An example of a typical maxillofacial fracture with sinus opacification can be seen in Figure 1.

Furthermore, Lewandowski, et al. performed a similar analysis of the "clear sinus sign" and found that a dedicated concurrent facial bone CT is not necessary for detection of sinus bone fracture as the maxillary sinuses are visualized on a routine head CT [7]. However, the current standard of care is to include a CT of the facial bones along with the head CT when there is suspicion of facial trauma. Therefore, we intend to expand the findings of the previous studies by analyzing the utility of the "clear sinus sign" using the current standard CT protocol for facial trauma. In doing so, we also evaluated the association of sinus opacification with other common facial fractures. Furthermore, our purpose is to reevaluate this association over a decade after the initial studies were performed. There has been a significant improvement in CT technology resulting in better image resolution, which could theoretically improve detection of subtle findings. In light of these recent advances, we aim to reassess whether the sensitivity and specificity of the clear sinus sign is improved or worsened.

## Materials and methods

This HIPPA compliant, retrospective designed study was approved by the Stony Brook University Committee on Research Involving Human Subjects Institutional Review Board (#496856-2). Informed patient consent was obtained at the time of treatment. Three readers with three years of radiology experience independently reviewed the study images and were blinded to the official radiology study report and clinical data for each patient. The readers were, however, aware of the purpose of the study.

The institutional electronic medical record was reviewed for patients’ clinical data, including history, physical exam, laboratory workup, potential procedures, and discharge information. CT scans of the facial bones without intravenous contrast performed between July 2012 and May 2013 were included in the study. All patients under the age of 18, postoperative CT exams, follow-up CT exams (e.g., prior history of known fractures, tumor follow-up, etc.), or CT scans of the facial bones performed for other indications (e.g., sinus disease, tonsillitis, foreign body evaluation, etc.) were excluded. A total of 764 consecutive CT scans of the facial bones without contrast were performed on patients who presented to the Emergency Department with a history of trauma between July 2012 and May 2013. Of these, 629 CT exams met the inclusion criteria. The CT exams were performed on a 320-detector row or 64-detector CT scanners. Coronal, sagittal, and 3D reformats were obtained for all studies as part of standard trauma CT facial bone protocol.  Imaging was performed on 320- and 64- detector row CT scanners. The 64-detector row helical CT scanner acquired images with an axial slice thickness of 0.625 mm; coronal and sagittal images were reconstructed with 3.0 mm slice thickness. Images on a 320-detector row system were volume scanned with an axial slice thickness of 0.5 mm and reconstructed images were 2.5 mm slice thickness in each plane.

The studies were then retrospectively analyzed by three reviewers for the presence of a facial bone fracture and/or fluid opacification of at least one paranasal sinus (as defined by either complete sinus opacification or an air-fluid level; circumferential mucosal thickening was considered an absence of fluid). The fractures were categorized and described, and a note made if the fractures involved the paranasal sinus walls. The studies were divided amongst the three reviewers, so each study was interpreted once.

The criteria for sinus opacification of the ethmoid sinuses is opacification of a single ethmoid cell, either anterior or posterior. Hounsfield units (HU) were measured and attenuation of greater than 20 HU was considered serosanguinous. The exception to this were cases in which the patient had stigmata of chronic rhinosinusitis including foci of calcification in the maxillary antrum and osteitis (suggested by cortical thickening and/or sclerosis) of the maxillary sinus walls. Additionally, if sinus contents were of heterogeneous density material, it was likely representative of infectious etiology. Furthermore, if there were lesions obstructing sinus drainage pathways, including polyps and neoplasms, these cases were also excluded [8-10].

## Results

**Figure 2:**
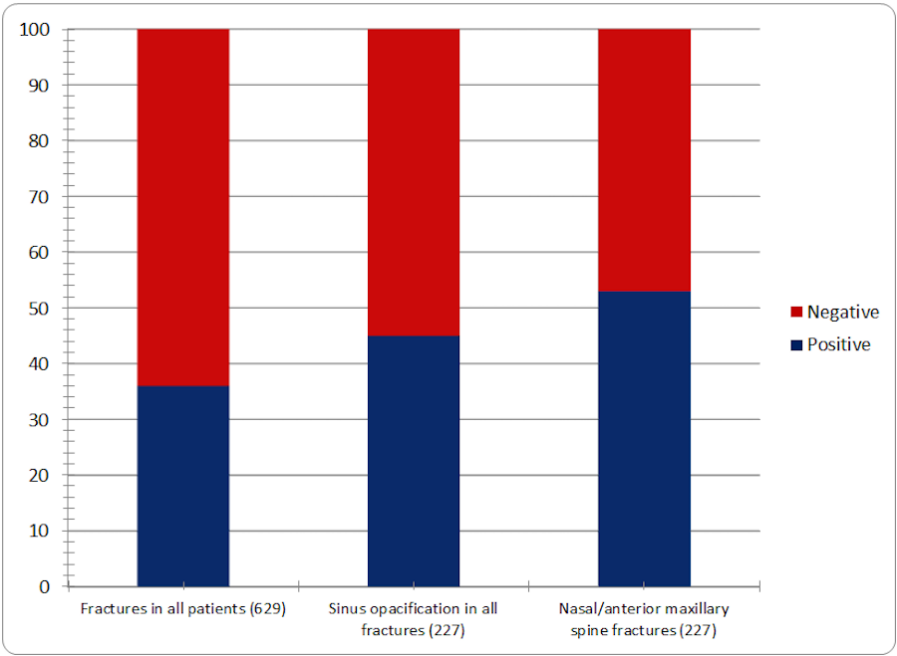
Percentage of positive fractures in all cases, sinus opacification in all cases, and nasal/anterior maxillary spine fractures out of all fractures Bar graph showing the percentage of positive fractures in all cases, sinus opacification in all cases, and nasal/anterior maxillary spine fractures out of all fractures with total fractures in each category indicated in parentheses. 36% of all studies contained facial bone fractures. 45% of facial bone fractures had sinus opacification. 53% of all fractures were nasal and anterior maxillary spine fractures.

Out of the 629 studies that were reviewed, 227 (36%) studies were positive for fracture, with 101 of these (45%) demonstrating sinus opacification. One hundred and nineteen of the 227 (53%) were isolated fractures of the nasal bones or anterior maxillary spine. These findings are shown in Figure 2.

**Figure 3:**
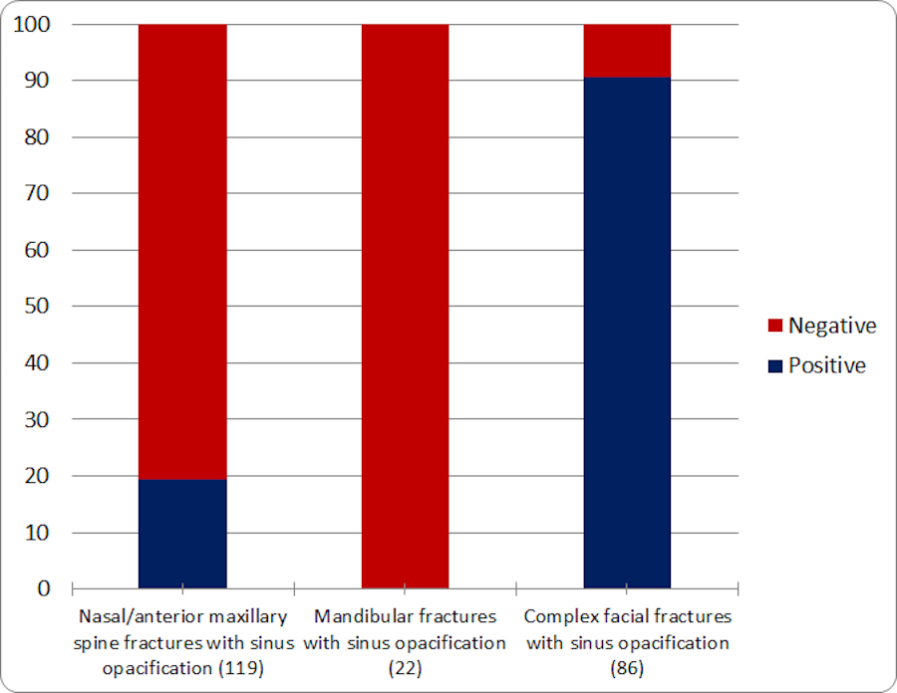
Percentage sinus opacification in different types of fractures Bar graph showing the percentage of different types of fractures with sinus opacification; total fractures in each category are indicated in parentheses. 91% of all complex facial fractures demonstrated sinus opacification, supporting the high specificity of sinus opacification for facial bone fracture. No mandibular fractures were associated with sinus opacification while only 19.3% of nasal bone and anterior maxillary spine fractures were associated with sinus opacification.

Of the 119 nasal bone or anterior maxillary spine fractures, 23 (19.3%) demonstrated sinus opacification. None of the 22 (10%) mandibular fractures had significant sinus fluid. Seventy-eight of the remaining 86 complex facial fractures (90.5%) demonstrated sinus opacification, which included zygomaticomaxillary complex (ZMC), orbital, and paranasal sinus fractures. These findings are summarized in Figure 3.

**Figure 4:**
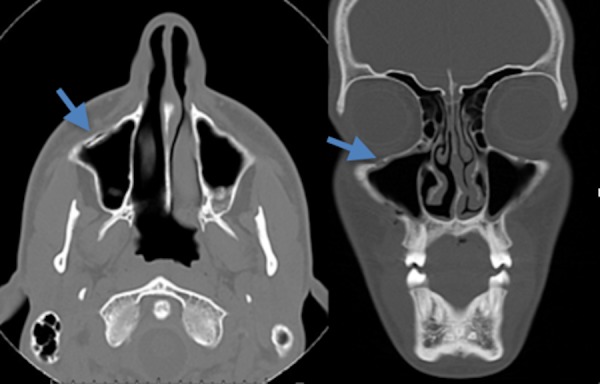
Minimally displaced maxillary sinus wall fracture without sinus opacification Axial (left) and coronal (right) CT scan of the facial bones demonstrates a non-displaced fracture (blue arrow) involving the anterior inferior wall of the right maxillary sinus. There is no associated fluid seen within the sinus.

Eight studies (9.3%) with these fractures demonstrated no appreciable sinus fluid. These included one maxillary fracture, one alveolar ridge fracture, a superior orbital wall fracture, a medial orbital wall fracture, a fracture of the sphenoid, a frontal calvarium fracture extending into the zygoma, an isolated zygomatic arch fracture, and a fracture of the anterior wall of the maxillary sinus. The medial orbital wall fracture is shown in Figure 4.

**Figure 5:**
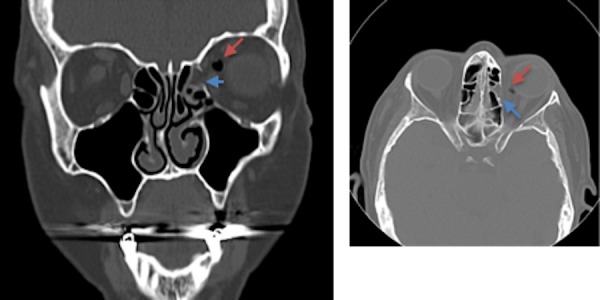
Coronal and axial CT of the facial bones demonstrating a non-displaced left orbital medial wall fracture Coronal (left) and axial (right) CT of the facial bones demonstrates a non-displaced left orbital medial wall fracture (blue arrow) resulting in intra-orbital air. There is minimal sinus opacification of the left medial ethmoid air cell without evidence of an air-fluid level present. An extra-sinus air locule (red arrow) is seen along the supromedial left orbit, likely post-traumatic in nature.

The maxillary sinus fracture is seen in Figure 5.

These were the only two fractures that involved the sinus walls without resulting sinus fluid. All eight of these patients’ charts were reviewed at their follow-up oral and maxillofacial surgery (OMFS) clinic visits. All fractures were treated conservatively, and none of the patients underwent surgical procedures to address these fractures.In one case of a right calvarial fracture, the patient required cranioplasty but the zygoma fracture was not treated.  

Fluid opacification of a sinus in the absence of fracture was found in only five of 402 patients (1.2%), p < 0.0001 by Fischer’s exact test. Presumably, these cases were due to incidentally discovered sinusitis.

## Discussion

With the annual US incidence of head injury reaching 100-300 per 100,000 persons, there is a tremendous volume of head CTs being performed every year. Facial trauma is often associated with additional cranial fractures and intracranial injury [11-14]. Therefore, radiologists are expected to interpret these CT scans rapidly because early identification and treatment have been shown to decrease complications [15-18]. The “clear sinus sign” is a well-known correlation used to rule out a maxillofacial fracture. It was initially evaluated by Lambert, et al. in 1997 and then again by Lewandowski, et al. in 2003. In this study, we re-analyzed the association of sinus opacification with paranasal sinus fracture. We also analyzed the association of the "clear sinus sign" with additional facial bone fractures, which are not included on routine head CT scans.

Compared to the previous studies, this analysis used improved CT technology and a larger sample size to re-evaluate the utility of the "clear sinus sign." The images had a thinner slice thickness of 0.625 millimeters (mm) and 3.0 mm versus 5.0 mm and 8.0 mm in the previous studies. With these higher resolution images, there is presumably less volume averaging, which can then more precisely demonstrate sinus opacification. Given these improvements, we did not find a major difference between our results and those of Lambert, et al. and Lewandowski, et al. Our data support the fact that the "clear sinus sign" is highly specific for facial bone fracture. However, when expanding the association of the "clear sinus sign" to other facial fractures, we found that it was less reliable.

More precisely, the "clear sinus sign" has 98.8% specificity for excluding facial fracture with a positive predictive value of 95.3%, but it is insensitive at 44.7%. Nasal bone and mandibular fractures cannot be ruled out in the setting of clear sinuses. However, for more complex facial fractures, such as ZMC, orbital, and sinus fractures, the lack of sinus fluid is significantly more sensitive at 91%. The negative predictive value for injuries requiring surgical intervention was 100%. While up to 9-10% of complex facial fractures may not have associated sinus opacification, these tend to be low-grade minimally displaced or non-displaced injuries which may be treated conservatively. This shows that sinus opacification is most useful in identifying severe facial injuries.

Furthermore, we would like to respond to Lewandowski and colleagues’ claim that a concurrent facial bone CT is not necessary for detection of sinus bone fracture. While we agree that, at the time of their study, maxillary and ethmoid fractures could be detected with certainty based on the presence of sinus opacification, we would like to point out that facial bone CT is useful in detecting additional facial fractures, which we found are not associated with sinus opacification, like nasal bone and mandibular fractures. Furthermore, with the current helical multidetector computed tomography (MDCT) acquisition, the detector row is placed at a higher pitch angle; therefore, the maxillary sinuses are often not included in the field of view of a non-contrast head CT. That is why our study analyzes “CT scan of the facial bones”, which is reflexively ordered by our ED physicians and trauma surgeons for any patient with suspected facial trauma. Lewandowski, et al. also stated that the addition of this protocol adds 15-30 minutes to the exam; however, this is not the case at our institution as acquisition time takes 1-3 minutes.

There are several limitations to our study. Because it is a retrospective analysis, this predisposes the study to selection bias and misclassification bias. A retrospective study is also dependent on the accuracy of other physicians’ assessment and documentation of the patients’ exposure to trauma and presenting symptoms. There was a small sample size, although larger than the previous two studies. Due to the small sample size, we cannot conclude with certainty that a lack of sinus fluid excludes the need for surgical intervention. Additionally, pediatric patients were excluded. Experimenter’s bias is another limitation of the study. Since the radiologists were aware of the purpose of the study, it may have increased the likelihood of making a positive finding. Furthermore, there is no independent gold standard for the presence of facial fracture and the findings. Sinus opacification and CT evidence of fracture are associated to an unknown degree in our hypothesis, this may lead to detection bias. It is possible that sinus opacification was “over-detected” due to the radiologists’ subconscious attempt to validate the aim of this study.

## Conclusions

We conclude that performing a facial bone CT scan, along with a routine head CT for patients with facial trauma, is appropriate in the current practice setting. Furthermore, our data supports that sinus opacification is still a strong correlate with complex facial bone fracture with improved CT imaging technology.

## References

[1] Sodickson A, Heitor O, Ledbetter S (2011). Spiral head CT in the evaluation of acute intracranial pathology: a pictorial essay. Emerg Radiol.

[2] Marx J, Hockberger R, Walls R, Adams J (2002). Emergency Medicine: Concepts and Clinical Practice. 5th ed.

[3] Haydel MJ, Preston CA, Mills TJ, Luber S, Blaudeau E, DeBlieux PM (2000). Indications for computed tomography in patients with minor head injury. N Engl J Med.

[4] Perry M, Morris C (2008). Advanced Trauma Life Support (ATLS) and facial trauma: can one size fit all? Part 2: ATLS, maxillofacial injuries and airway management dilemmas. Int J Oral Maxillofac Surg.

[5] Wang X, You JJ ( 2013). Head CT for nontrauma patients in the emergency department: clinical predictors of abnormal findings. Radiology.

[6] Lambert DM, Mirvis SE, Shanmuganathan K, Tilghman DL (1997). Computed tomography exclusion of osseous paranasal sinus injury in blunt trauma patients: the "clear sinus" sign. J Oral Maxillofac Surg.

[7] Lewandowski RJ, Rhodes CA, McCarroll K, Hefner L (2004). Role of routine nonenhanced head computed tomography scan in excluding orbital, maxillary, or zygomatic fractures secondary to blunt head trauma. Emerg Radiol.

[8] Khalid AN, Hunt J, Perloff JR, Kennedy DW (2002). The role of bone in chronic rhinosinusitis. Laryngoscope.

[9] Nair S (2009). Correlation between symptoms and radiological findings in patients of chronic rhinosinusitis: a modified radiological typing system. Rhinology.

[10] Bhattacharyya N, Fried MP (2003). The accuracy of computed tomography in the diagnosis of chronic rhinosinusitis. Laryngoscope.

[11] Zacharia TT, Nguyen DT (2010). Subtle pathology detection with multidetector row coronal and sagittal CT reformations in acute head trauma. Emerg Radiol.

[12] Huikeshoven M, Wong N, Bush KL (2014). Nasofrontal outflow tract visibility in computed tomography imaging of frontal sinus fractures. Cranialmaxillofac Trauma Reconstr.

[13] Yakirevitch A, Bedrin L, Alon EE, Yoffe T, Wolf M, Yahalom R (2013). Relation between preoperative computed tomographic criteria of injury to the nasofrontal outflow tract and operative findings in fractures of the frontal sinus. Br J Oral Maxillofac Surg.

[14] Avery LL, Susarla SM, Novelline RA (2011). Multidetector and three-dimensional CT evaluation of the patient with maxillofacial injury. Radiol Clin North Am.

[15] Chen KT, Chen CT, Mardini S, Tsay PK, Chen YR (2006). Frontal sinus fractures: A treatment algorithm and assessment of outcomes based on 78 clinical cases. Plast Reconstr Surg.

[16] Fox PM, Garza R, Dusch M, Hwang PH, Girod S (2014). Management of frontal sinus fractures: treatment modality changes at a level I trauma center. J Craniofac Surg.

[17] Kellman R, Goyal P (2014). Managing the frontal sinus in the endoscopic age: has the endoscope changed the algorithm?. Craniomaxillofac Trauma Reconstr.

[18] Winegar BA, Murillo H, Tantiwongkosi B (2013). Spectrum of critical imaging findings in complex facial skeletal trauma. Radiographics.

